# Chronic Heart Failure Management: Monitoring Patients and Intercepting Exacerbations

**DOI:** 10.31083/j.rcm2407208

**Published:** 2023-07-17

**Authors:** Gianfranco Piccirillo, Federica Moscucci, Susanna Sciomer, Damiano Magrì

**Affiliations:** ^1^Dipartimento di Scienze Cliniche, Internistiche, Anestesiologiche e Cardiovascolari, Policlinico Umberto I, “Sapienza'' University of Rome, 00161 Rome, Italy; ^2^Department of Internal Medicine and Medical Specialties, Policlinico Umberto I, 00161 Rome, Italy; ^3^Dipartimento di Medicina Clinica e Molecolare, S. Andrea Hospital, “Sapienza'' University of Rome, 00189 Rome, Italy

**Keywords:** heart failure, prognosis, repolarization, sex/gender peculiarities, ECG markers, bioimpedance

## Abstract

Despite significant progress in the field of therapy and management, chronic heart failure (CHF) still remains one of the most common causes of morbidity and mortality, especially among the elderly in Western countries. In particular, frequent episodes of decompensation and, consequently, repeated hospitalizations represent an unsustainable burden for national health systems and the cause of worsening quality of life. CHF is more prevalent in elderly women, who often have “peculiar” clinical characteristics and a more preserved ejection fraction caused by endothelial dysfunction and micro-vessel damage. At the moment, noninvasive technologies that are able to remotely monitor these patients are not widely available yet, and clinical trials are underway to evaluate invasive remote sensors. Unfortunately, implantable devices for identifying decompensation are not the most practical solution in the majority of of patients with chronic heart failure. In particular, they are hypothesized to have the possibility of monitoring patients by pro-B-type natriuretic peptide, ventricular repolarization variability, and bioimpedance cardiography at the first point of care, but new technology and clinical trials must be planned to address the development and spread of these emergent possibilities.

## 1. Introduction

Chronic heart failure (CHF) is becoming a real social emergency both in Western countries and in those with emerging economies. In fact, with the progressive increase in population aging, the prevalence of CHF in elderly patients is higher than 10% [1]. It is interesting to emphasize in the COVID-19 pandemic era that re-acutizations in CHF are frequent and the deadliest comorbidities [2]. Although enormous progress has been obtained in CHF treatments in the last two decades, and despite optimal medical therapy or the use of the most modern implantable devices, decompensated CHF is a critical health system issue. For example, the in-hospital mortality rate is about 4%, and after discharge, mortality tends to worsen; in fact, the 30-day, 1-year and 5-year mortality rates are 10%, 22%, and 42%, respectively [3]. To stratify the mortality and mobility risks, the research is currently focused on new, simple, noninvasive, easily repeatable, and inexpensive markers of the decompensation of CHF, but above all, it is necessary to validate them in homogenous groups of CHF patients. To obtain these data, recently, the European Heart Society [4], following the indications of other scientific societies [5], redefined this syndrome, historically based on systolic function. From this point of view, it is possible to distinguish three pathophysiologic, hemodynamic conditions with different systolic functions based on the left ventricular ejection fraction (LVEF): symptomatic subjects with heart failure with heart failure with reduced ejection fraction (HFrEF), those with heart failure with mildly reduced ejection fraction (HFmrEF), and finally, symptomatic subjects with heart failure preserved ejection fraction (HFpEF). The ejection fractions are ≤40% in subjects with HFrEF, between 41 and 49% in the HFmrEF subjects, and ≥50% in those with HFpEF [4, 5]. Likely, the initial mechanism in HFpEF patients is a predominant diastolic dysfunction induced by left ventricular hypertrophy and/or chronic myocardial ischemia; nevertheless, the precise mechanisms still remain controversial.

This redefinition aims to obtain more homogeneous data to improve the risk stratification of mortality and morbidity and to promote comparable therapeutic clinical trials able to induce an increased knowledge in this field. In fact, almost all clinical trials have focused on treatment for patients with systolic severe dysfunction, especially even with worse LVEF ≤35%; moreover, all of the available studies on patients with LVEF >40% are mostly incomparable for the different classifications of CHF [4].

Previously, four stages to classify the CHF was proposed as stages A, B, C, and D, from risk of heart failure (HF) (stage A), to advanced HF (stage D) [6].

Before this classification into four stages, the functional class of the New York Heart Association (NYHA) was introduced, based on the severity of symptoms [7].

Therefore, the aim of this review is to evaluate noninvasive markers that are able to identify early possible re-exacerbations in patients with advanced CHF.

## 2. Pathophysiological and Clinical Aspects of Chronic Heart Failure

**Fig. 1.**
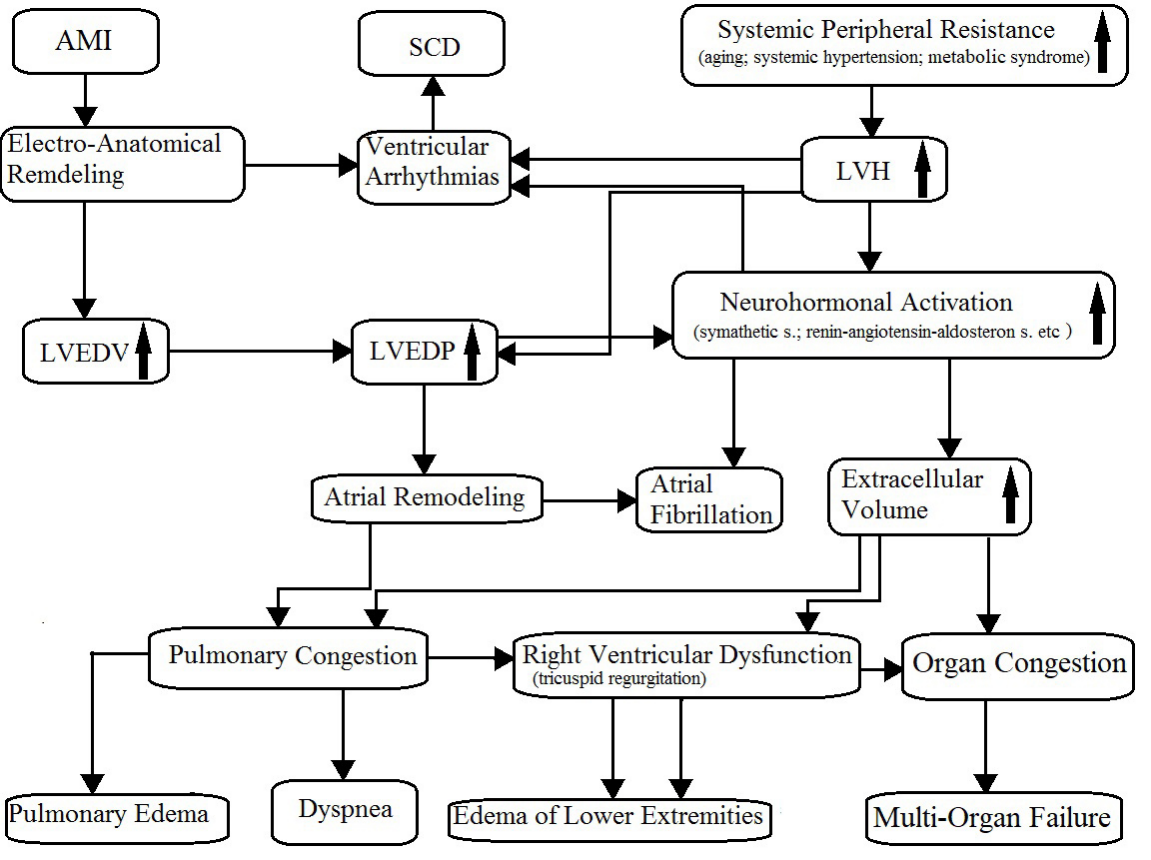
**Pathophysiology of chronic heart failure**. AMI, acute myocardial infarction; SCD, sudden cardiac death; LVH, left ventricular hypertrophy; LVEDV, left ventricular end-diastolic volume; LVEDP, left ventricular end-diastolic pressure. The arrows mean “increase”.

As is known, CHF is a progressive disease characterized by a close pathophysiologic relationship between systolic and diastolic dysfunction because of the increase in the left ventricular end-diastolic pressure (LVEDP), also known as filling pressure. In HFrEF or HFmrEF, the increase in LVEDP originates mostly after an acute myocardial infarction or, less often, from other cardiomyopathies. On the other hand, the HFpEF is a direct consequence of left ventricular hypertrophy most frequently due to systemic hypertension or hypertrophic cardiomyopathy. In HFpEF patients, the left ventricular end-diastolic volume (LVEDV) can be normal; conversely, among HFrEF and HFmrEF patients, it is mildly or severely increased. The major consequences of an increase in LVEDP are atrial enlargement, pulmonary congestion, renin–angiotensin–aldosterone system imbalance and sympathetic activation. As result, fluid retention with edema, sinus tachycardia or atrial and ventricular arrhythmias (palpitations), and dyspnea (Fig. 1) could occur. Then, dyspnea and edema of the lower extremities are the direct consequences of pulmonary congestion and fluid retention, respectively; typical consequences of reduced systolic function and peripheral hypoperfusion are fatigue, weakness (asthenia), lightheadedness, and nocturia.

Notably, it should be emphasized that some authors have demonstrated that pulmonary artery wedge pressure (PAWP) is more closely related to outcome in HFpEF than LVEDP [8]. In the study by [8], the authors observed that the carbon monoxide-diffusing capacity (DLCO) was the only parameter that was independently correlated to the pressure difference between the PAWP and the LVEDP, concluding that both low DLCO and the pressure gradient between the PAWP and the LVEDP reflect thickening of the alveolar–capillary membrane due to chronic congestion. Therefore, according to the authors, both parameters are associated with the disease severity and should be addressed in future large-scale studies [8].

**Fig. 2.**
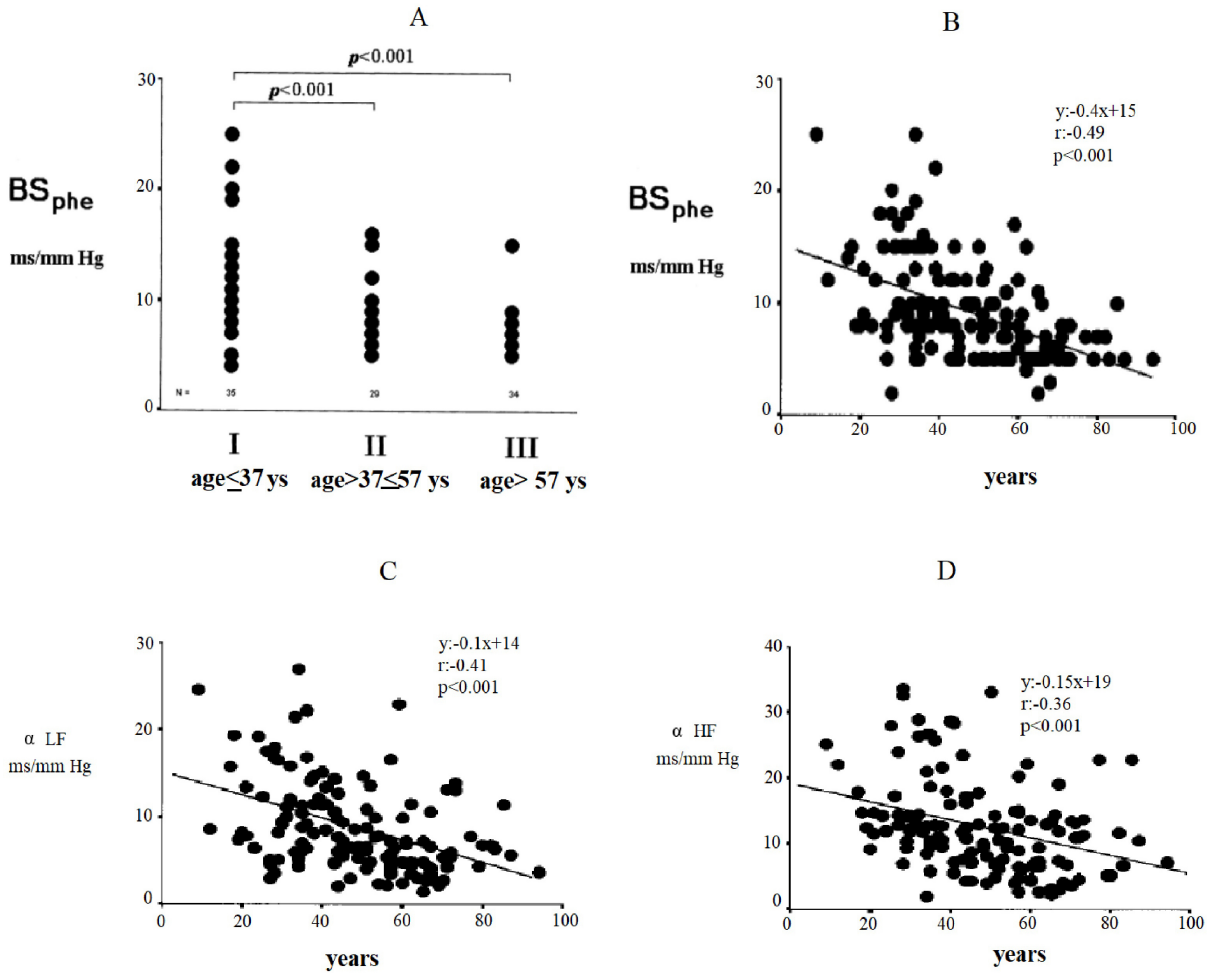
**Changes in baroreflex sensitivity by the phenylephrine method (${\mathrm{BS}}_{\text{𝐩𝐡𝐞}}$)**. Subjects grouped for tertiles of age (ANOVA and Bonferroni tests). (A) Relationship between age and baroreflex sensitivity with phenylephrine method. (B) Relationship between age and α low-frequency (LF). (C) Age and apha high-frequency (HF). (D) Age and sympathetic of vagal etc. AVOVA, ANalysis Of VAriance.

In this pathological situation, sympathetic overstimulation is not counterbalanced by sufficient vagal activation. As a result, the vagal sinus control is reduced and the baroreflex sensitivity is blunted [9, 10, 11]. This phenomenon has been used to stratify the mortality risk in post-myocardial infarction [12, 13, 14, 15, 16] and CHF [17] patients. In fact, it is possible to observe a reduction in the heart rate variability in all of the electrocardiographic (ECG) spectral components [9, 18, 19] and an increase in the temporal dispersion of left ventricular repolarization in both post-myocardial infarction and CHF [19, 20, 21, 22, 23, 24]. However, the final result was a reduction in vagal sinus control, and the vagal nerve activity recorded directly from the left thoracic vagal nerve increased in experimental CHF [25, 26], as shown in Fig. 2. In this figure, changes in baroreflex sensitivity are reported based on the phenylephrine method (${\mathrm{BS}}_{\text{phe}}$) (Panel A), grouping patients by age tertiles. The relationship between age and baroreflex sensitivity (Panel B) is shown, as is the relationship between age and α low-frequency (α LF) (Panel C) or α high-frequency (α HF) (Panel D), namely the sympathetic or vagal components of baroreflex sensitivity calculated by power spectral analysis.

Probably, CHF prevents the conversion of vagal nerve activity in a reduced chronotropic response at the sinus node level [25, 27]. In other words, CHF reduces the vagal nerve capacity to modulate the respiratory oscillation of the heart rate at the sinus node level. Normally, in fact, the autonomic nervous system modulates the heart rate respiratory oscillation, also known as “respiratory sinus arrhythmia”. On the contrary, during CHF, the autonomic nervous system becomes unable to modulate the heart rate variability, and the loss of this physiological property is associated with the severity of symptoms, sympathetic hyperactivity, and an increase in cardiovascular mortality and the risk of sudden death.

### 2.1 Acute Exacerbation of CHF

**Fig. 3.**
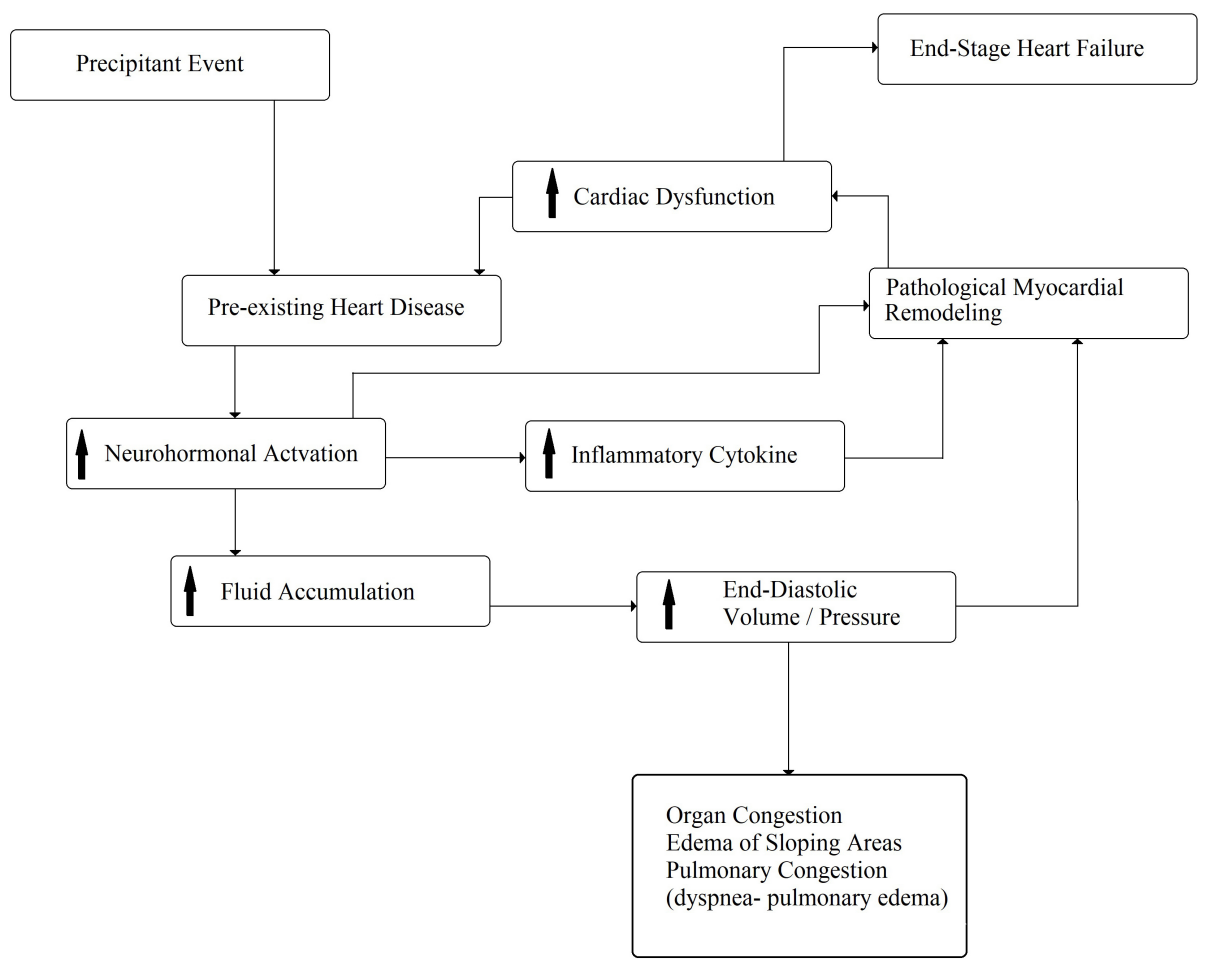
**Pathophysiology of acute decompensated chronic heart failure**. The arrows mean “increase”.

Despite the progress of knowledge and the use of new drugs and devices, as previously emphasized, CHF remains a progressive, clinical syndrome with recurrent acute decompensations and severe prognoses. In fact, the hospital readmission rates of CHF patients are about 24%, 30%, and 50% within 30 days, 3 months, and 6 months, respectively. The major causes of these rehospitalizations are characterized by respiratory symptoms or fluid retention and pulmonary congestion, with the worsening of pre-existing dyspnea and/or systemic edema [3]. The precipitating factors of acute decompensated CHF are numerous, but the most important factors are concurrent infections, atrial fibrillation, uncontrolled arterial hypertension, acute coronary syndrome, and low compliance with the drugs or dietary prescriptions; however, at least 50% of cases remain unknown, and, in most cases, multiple factors can be identified [28, 29]. Some studies have reported that the mortality rate is related to the frequency of decompensated episodes [30, 31]. It was hypothesized that each acute episode increases the mortality rate because the pre-existent structural heart disease tends to worsen. This phenomenon seems to be associated with an acceleration of pathologic remodeling (Fig. 3), demonstrated by a transient increase in ultra-sensitive troponin I and extracellular matrix turnover (matrix metalloprotease 2, matrix metalloprotease 1, and procollagen type III N-terminal peptides) [32, 33].

Especially in acute decompensation, a key role could be played by the inflammatory state. For example, in some clinical trials on CHF, the prevalence of inflammation was about 57% [34], which became more and more relevant with the aging process [35], systolic or diastolic dysfunction [36], and decompensation severity [35]. For instance, some authors recently reported that in a cohort of in-hospital elderly (median age of 85 years) patients with decompensated CHF [37], the prevalence of the inflammatory state was about 86% (median high sensitivity C reactive proteine (hsCRP): 3.46 mg/dL). Regarding the pathophysiologic mechanism, it was reported that the pro-inflammatory cytokine interleukin-1 and tumor necrosis factor-α (TNF-α) were able to induce systolic and diastolic dysfunction, and TNF-α was specifically able to promote pathological myocardial remodeling (Fig. 3) [38]. Despite these experimental data and a reduction in blood inflammatory biomarkers, the administration of anti-cytokine and anti-inflammatory therapies in CHF patients reported unsatisfactory results [37]. This could suggest that immune involvement should be considered as an epiphenomenon rather than a central pathophysiologic mechanism of CHF.

### 2.2 CHF: The Gender Matter

As already stated, HF is among the most frequent causes of hospitalization in the medical and cardiology departments in European Countries [39], presenting a mortality rate of 40% within 1 year after the first hospitalization and a 5-year survival rate between 25 and 38%, based on sex, age, comorbidities, and the severity of the HF itself.

Notoriously classified on the basis of left ventricular function [4], HF is often a consequence of coexisting ischemic, hypertensive, diabetic, or other heart disease, which has resulted in profound changes in the contractile capacity of the heart and in the patient’s hemodynamics [40, 41, 42, 43]. As evidenced by many recent studies, symptoms of HF in women are often vague and unfortunately not recognized as “typical” [44, 45]. The physiopathology of the damage is profoundly different; endothelial dysfunction, micro-vessel damage [46], and comorbidities (e.g., diabetes mellitus, systemic arterial hypertension, autoimmune diseases, and drug cardiotoxicity) are, in women, frequently associated with a more preserved ejection fraction than in men [40]. If the main cause of HF in men is ischemic heart disease caused by obstructive coronary artery disease, in women, significant microcirculation and small vessel ischemic damage (myocardial infarction with no obstructive coronary arteries, myocardial infarction with non-obstructive coronary arteries (MINOCA)) is more commonly observed [47, 48]. For this reason, symptoms, clinical course, and prognosis are peculiar, as is the therapeutic response [49]. Symptoms of HF in women are often vague and considered “atypical”, with frequent delays in diagnosis and belated arrival to the doctor’s attention. For this reason, women are often much older at the time of diagnosis, and they usually show a higher degree of co-pathology and polypharmacy than men, with a higher risk of the drug’s side effects and poor adherence to therapies [40]. In this situation, female patients are highly exposed to the risk of iatrogenic damage, poor compensation, and therapeutic failure. Age is also often a limiting factor for enrollment in clinical trials, which, therefore, frequently has a gender bias [40].

It has also been shown that female patients with heart failure, while significantly benefiting from the implantation of devices (pacemakers and implantable defibrillators) or resynchronization therapy, rarely receive an indication for this type of intervention [50].

Prognostic evaluation is also particularly difficult in the female population affected by heart failure. The Heart Failure Survival Score [51], Seattle Heart Failure Model [52], Meta-Analysis Global Group in Chronic Heart Failure [53], and Metabolic Exercise Cardiac Kidney Index [54] Score, used for the risk stratification of death or the need for the urgent cardiac transplantation of patients suffering from heart failure, do not allow for a sex-specific evaluation, even though substantial discrepancies between the two sexes have been identified in some studies [55, 56]. In fact, compared to scores describing severe disease, women often have better prognoses. This could depend on the underlying differences in the pathophysiology of cardiac damage that determine a serious and more disabling clinical presentation, which may not correspond to an equally poor prognosis.

However, it should be reiterated that HF ​​in elderly women causes an extremely significant proportion of repeated hospitalizations (“revolving door”), with episodes of acute HF (dyspnoea, acute pulmonary edema) at increasingly shorter intervals, until death [57]. This not only results in high management costs but also has a severe impact on the quality of life.

Advanced age at diagnosis and the difficulty of stratifying the risk for the decompensated patient are reasons why female patients are historically less eligible for heart transplantation [58].

This evidence clearly demonstrates how a gender-specific approach to this particularly complex condition is absolutely indispensable and necessary [45] in order to guarantee target diagnoses, treatments, and therapies to reduce morbidity, mortality, and management costs (Table 1).

**Table 1.** **Peculiarities of chronic heart failure among women**.

Specific characteristics of chronic heart failure in women	
Different pathophysiology	Endothelial dysfunction; micro-vessel damage (diabetes, arterial hypertension, estrogen depletion after menopause)
Most common symptoms	More severe weakness; reduced exercise tolerance; diaphoresis; more pronounced dyspnea; precordial palpitations
Diagnostic delay determining a later-in-life diagnosis	High degree of polypathology, polypharmacy, and iatrogenic damage; reduced access to the heart transplant; exclusion from clinical trials
Difficult prognostic evaluation	Scores/risk charts formulated on male models, and therefore, not designed and studied for women; no score currently takes into account sex-specific risk factors
High “revolving door” risk	High costs for health systems; reduced patient quality of life

Specific diagnostic and therapeutic interventions, together with an education program to enhance clinicians’ and women’s awareness, are desirable [59]. These would make it possible to reduce repeated hospitalizations in the health sector, reduce mortality and morbidity, and, at the same time, improve the quality of life in the female population, which, historically, has been and is considered protected from cardiovascular disease and its consequences.

### 2.3 Common Biomarkers of CHF

All biomarkers should be designed, experimented, and implemented to simplify the diagnostic process. In fact, the essential features of biomarkers are the highest levels of specificity, sensitivity, and predictive values. Clearly, these characteristics are not enough nowadays. In fact, a specific and modern CHF biomarker should also present other features: rapid measurement, noninvasive, inexpensive, easily collectible, repeatable, and reproducible. Finally, it should be able to improve the diagnostic process, monitor the progress of the disease during treatments, and be a reliable prognosis tool at the same time.

**Fig. 4.**
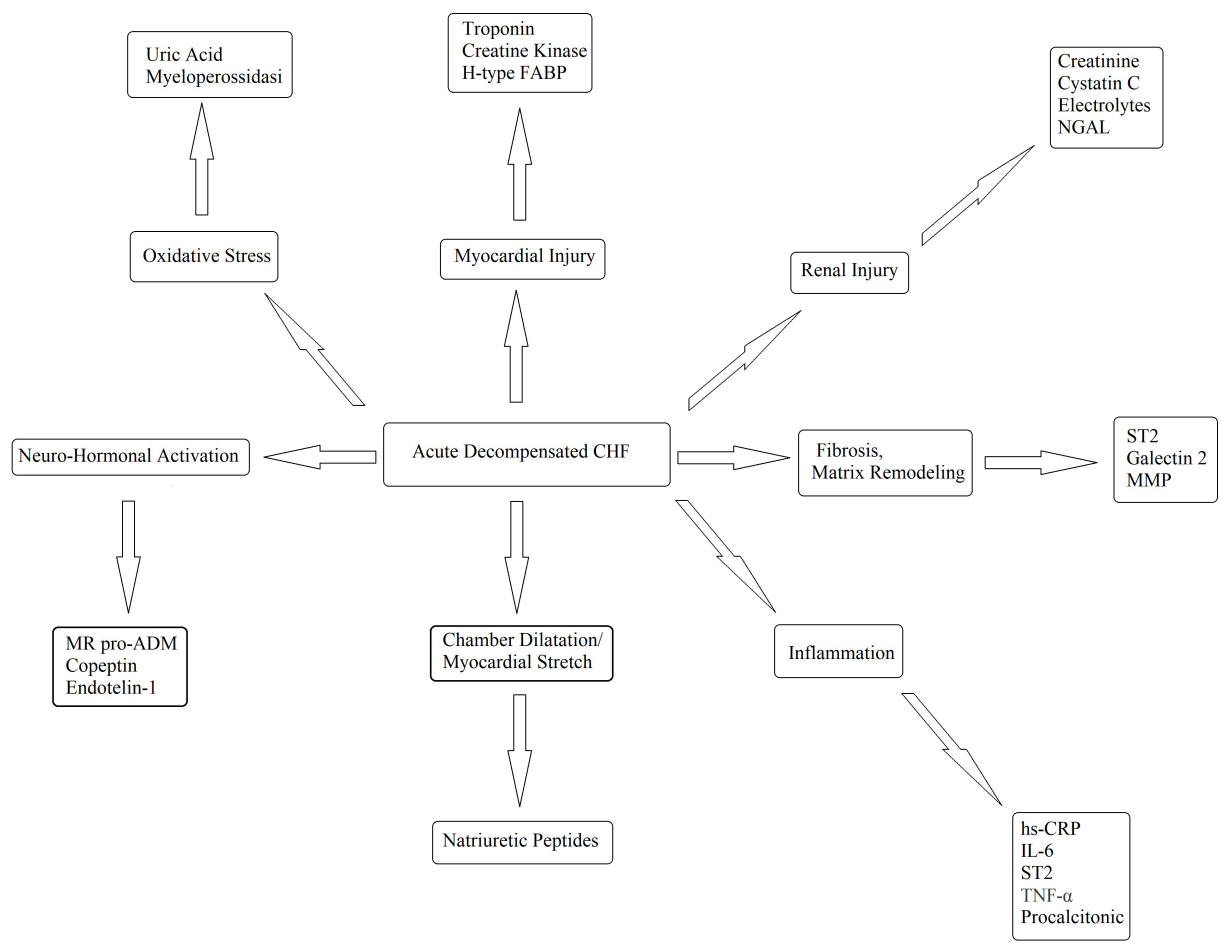
**Potential biomarkers in the diagnosis and management of decompensated CHF**. FABP, fatty acid-binding protein; hsCRP, high-sensitivity C-reaction protein; IL, interleukin; MMP, matrix metalloproteinase; NGAL, neutrophil gelatinase-associated lipocalin; ST2, suppressor of tumourigenicity-2; TNF, tumor necrosis factor; CHF, chronic heart failure; MR pro ADM, mid-regional proadrenomedullin; IL-6, Interleuki-6.

**Fig. 5.**
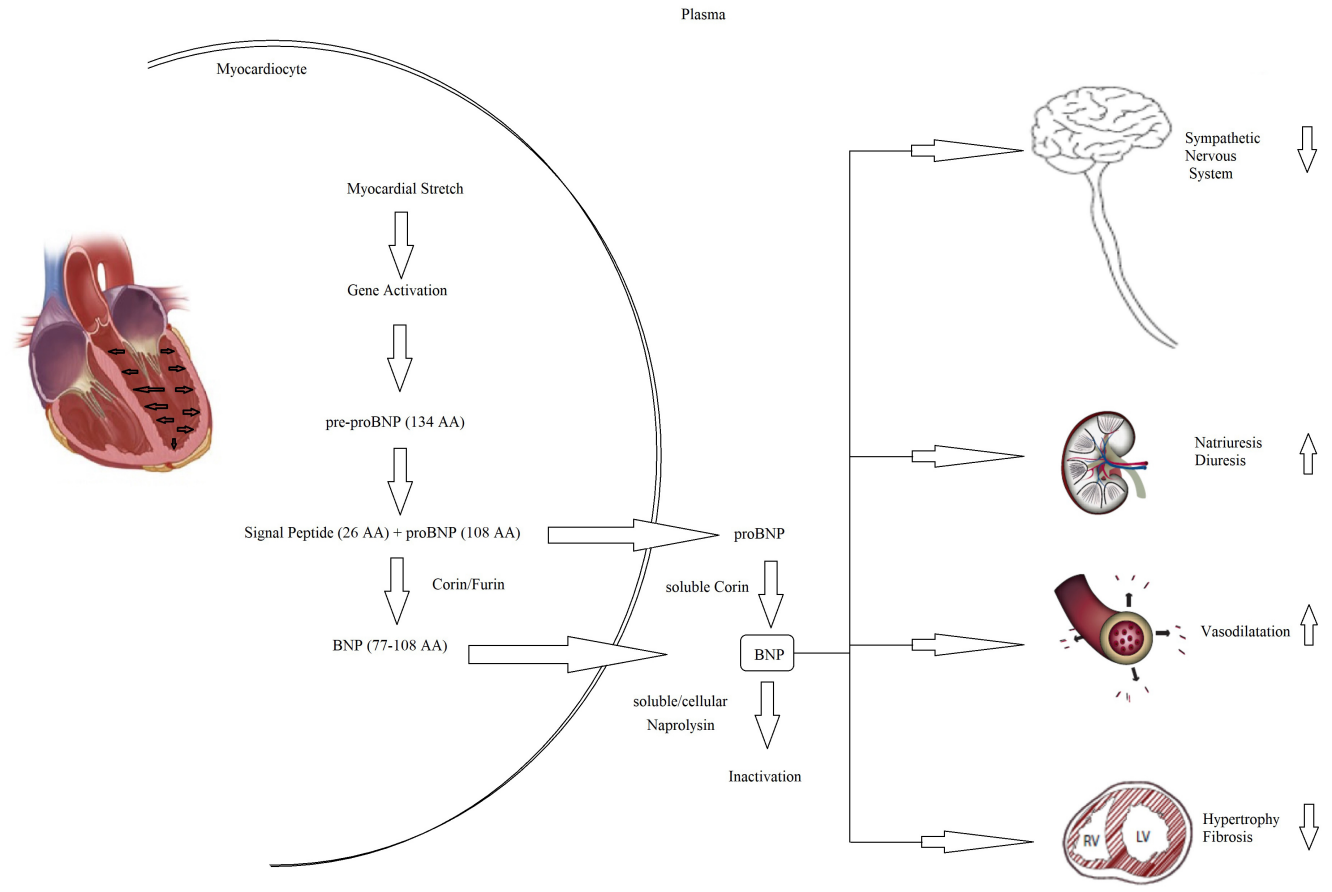
**Pathophysiology of NT-proBNP and BNP**. BNP, B-type natriuretic peptide; NT-proBNP, N-terminal pro-B-type natriuretic peptide; AA, aminacyd.

Every possible biomarker must have a solid pathophysiologic basis and, as CHF is a complex syndrome, many biomarkers have been proposed and tested (Fig. 4) [60]. Although countless possible biomarkers based on different pathophysiologic substrates have been studied, natriuretic peptides have been the most extensively studied [60, 61]. Natriuretic peptides are a group of similar peptides of atrial, ventricular, and endothelial origin, but the most studied, as CHF markers, are B-type natriuretic peptide (BNP) and its prohormone, namely, N-terminal pro-B-type natriuretic peptide (NT-proBNP) (Fig. 5). The biological activity of natriuretic peptides is to increase diuresis, natriuresis, and vasodilatation and to inhibit the renin–angiotensin–aldosterone and the sympathetic system’s overactivity (Fig. 5). Both BNP and NT-proBNP are produced by myocytes as a consequence of myocardial wall stretch and are specifically induced by a pathological increase in LVEDV/P. NT-proBNP and BNP are cleaved from pre-proBNP, and NT-proBNP is biologically inactive but has a more stable status. In fact, the half-life of BNP is 20 minutes, while NT-proBNP has a half-life between 60 and 120 minutes [60, 61, 62], with a ratio higher than 1:6 [63]. The blood kinetics of BNP and NT-proBNP are influenced by CHF exacerbation but also by aging, gender, renal function, obesity, genetic factors, comorbidities, obesity, and LVEDV/P.

Iwanaga Y *et al*. [64] showed in a previous study that plasma BNP levels reflect left ventricular end diastolic wall stress (EDWS) more than any other parameter previously reported, not only in patients with HFrEF but also in patients with HFpEF. The relationship of left ventricular EDWS to plasma BNP may be an important parameter to consider in HFpEF patients, providing a better fundamental understanding of the individual heterogeneity among BNP levels and their clinical utility in the diagnosis and management of HF (especially in specific groups: e.g., elderly, women).

Many clinical trials have assessed the role of BNP and NT-proBNP in the diagnosis, management, and prognosis of HF. The end-point of these studies was to assess the correct HF diagnosis in patients with breathlessness. Particularly, the first studies conducted on these issues were the Breathing Not Properly Study for BNP and the ProBNP Investigation of Dyspnea in the Emergency Department (PRIDE) for NT-proBNP [65, 66]. In both, the natriuretic peptides were the best significant predictors of the final diagnosis [65, 66]. Although they have been considered in the clinical practice guideline as Class I and level of evidence A, some critical issues remain unresolved [4, 67]. In fact, as abovementioned, some comorbidities (diabetes, atrial fibrillation, obesity, renal insufficiency), gender, and age have a not negligible role on the plasmatic levels of both natriuretic peptides. For example, the International Collaborative of NT-proBNP: Re-evaluation of Acute Diagnostic Cut-Offs in Emergency Department Study (ICON-Reloaded) tried to dampen the age influence on natriuretic peptides [38]. In fact, the authors stratified for age three different NT-proBNP cut-offs (<50 years: <450 pg/dL; 50–75 years: <900 pg/dL; >75 years: <1800 pg/mL), but even in this way, the sensitivity and specificities were reduced according to aging (sensitivities: <50 years: 85.7%, 50–75 years: 79.3%, and >75 years: 75.9%; specificities <50 years: 93.9%, 50–75 years: 84.0%, and >75 years: 75.0) [68]. Thus, the cut-offs for the diagnosis of HF should be increased with the function of age. Nevertheless, the authors observed reductions in sensitivity and specificity, likely due to renal insufficiency, which is frequent among the elderly. In fact, a correction based on the glomerular filtration rate (GFR) was proposed: for a GFR <60 mL/min/1.73 $m^{2}$ a cut-off of 1200 pg/mL, resulting in sensitivity and specificity of 89% and 72%, respectively [68]. Obviously, among very old CHF patients, aging and a low GFR can coexist, becoming confounding factors and negatively affecting the diagnostic power of the NT-proBNP.

Another serum biomarker, specifically studied for HF diagnosis, was the highly sensitive troponin (hs-Tn). Troponins are proteins involved in skeletal and cardiac muscle contraction. Specific cardiac isoforms were found to be increased not only in myocardial necrosis but even in decompensated CHF, so they could be considered a marker of myocardial necrosis in acute myocardial infarction and also a marker of myocardial wall stress during acute decompensated CHF injury. In the Acute Decompensated Heart Failure National Registry (ADHERE), the authors reported an increase in in-hospital mortality risk associated with higher levels of hs-Tn [69]. These data have been confirmed in further trials on acute decompensated CHF, about the global [70, 71] and cardiovascular mortality [72]. Finally, other markers of hypertrophy, remodeling, and inflammation (Fig. 4) have been tested in many studies with different end-points, demonstrating more usefulness for interpreting the different pathophysiologic aspects than making a diagnosis and risking stratification, so they are not routinely used.

**Fig. 6.**
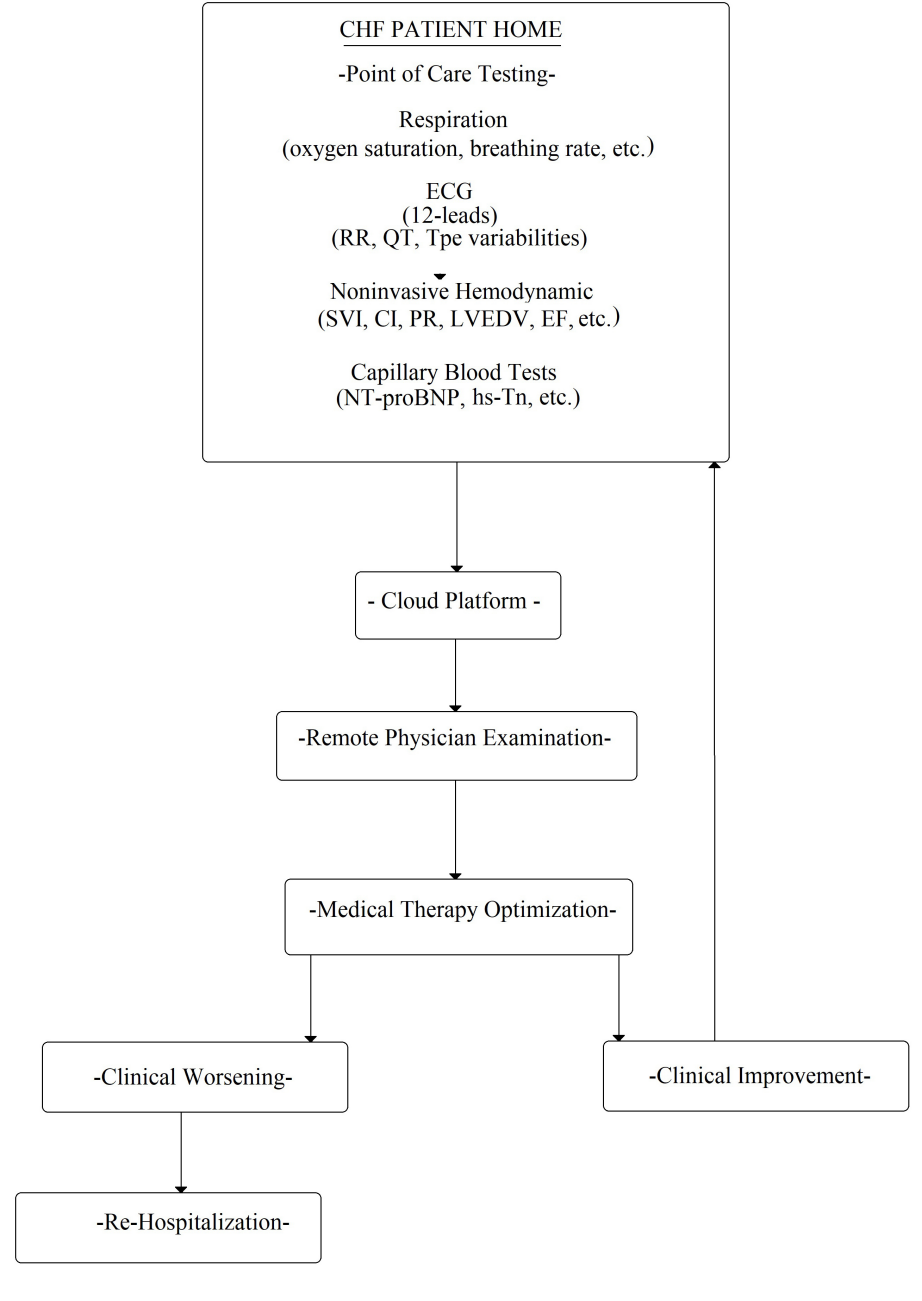
**Flowchart of possible CHF patient management at home**. ECG, electrocardiogram; RR, wave interval; QT, QT interval; Tpe variabilities, Tpeak-end variabilities; SVI, stroke volume index; CI, cardiac index; PR, pulse rate; LVEDV, left ventricle end dyastolic volume; EF, ejection fraction; NT-proBNP, N-terminal pro-B-type natriuretic peptide; hs-Tn, high sensitivity Troponine.

Finally, the possibility of obtaining the dosage of a marker from the capillary blood test is of great clinical relevance (as the glucose from the fingertip in diabetic patients). In other words, this could allow for the analysis of the biomarker in different settings: at the point-of-care or at the patient’s home, to avoid or choose hospitalization. In fact, nowadays, specific instruments are commercially available to measure NT-proBNP [73] and troponin [74, 75, 76], which are detected from a capillary blood sample using a single-use test strip. Similar to capillary glucose test, the blood drop test should be performed by the patients themselves. This hypothetical procedure could allow for the patient’s follow-up at home (Fig. 6).

### 2.4 Possible Electrical CHF Markers

The greatest challenge in CHF in modern medicine is patient management. In fact, the acute symptom re-exacerbations and the consequent rehospitalizations are a serious concern for the health system. As CHF mortality has been reduced and life expectancy has increased, the prevalence of failing to heal has grown. Moreover, the more the survival rate increases, the more re-exacerbations, rehospitalizations, and healthcare costs will do. At present, despite new promising drugs and devices, acute decompensated CHF remains an unsolved issue. Thus, to avoid repeated hospitalizations, telemonitoring CHF patients could be a key point. This monitoring should be available especially at home, with possible remote access by physicians. In this way, the drug therapy could be changed and adapted, possibly avoiding re-exacerbation, rehospitalization, and mortality (Fig. 6). The ideal candidates among noninvasive CHF markers are electrical signals, offering standard electrocardiographic and hemodynamic (bioimpedance cardiography) information with numerous advantages. In fact, they are simple to detect, but also repeatable, transmissible, and inexpensive. It is possible to distinguish two kinds of signals: those based on an ECG in twelve leads and those based on the RR interval or repolarization variabilities. Obviously, a standard ECG is essential in the early diagnosis of arrhythmias and myocardial ischemia or necrosis, but it could offer other significant benefits [77]. All considered, an ECG serial analysis based on the variability of different QRS intervals could offer new insight into CHF exacerbation phenomena. In particular, short-period RR variability [18, 25, 78], available with many interpretative algorithms and methods, could be used to evaluate the changes in autonomic control during and before a CHF decompensation, leading to drug therapy changes, in order to reduce sympathetic overdrive [11, 20, 79, 80, 81]. A more modern alternative to RR variability is represented by the QT variability. Obtained by different formulas [20], this is a short-term marker of the temporal dispersion of left ventricular repolarization. Calculated by 5-minute ECG recordings, it expresses the temporal inhomogeneity of myocardial repolarization and, especially in post-ischemic [82] or primitive dilative cardiomyopathy [20, 83], it is a marker of sudden cardiac death or cardiovascular mortality [20, 21, 84]. In particular, the most used repolarization marker is the QT variability index (QTVI). QTVI was introduced by Berger in 1997; the author stated the QTVI as the logarithm of the ratio between QT variability (QT variance/QT ${\mathrm{mean}}^{2}$) and RR variability (RR variance/RR ${\mathrm{mean}}^{2}$) [85]. Other studies have considered only a numerator of the aforementioned formula (QT variance/QT ${\mathrm{mean}}^{2}$) [20, 21, 86] or the standard deviation of the mean of QT intervals (${\mathrm{QT}}_{\text{SD}}$) [20, 86, 87, 88, 89, 90].

A meta-analysis based on almost 2000 healthy subjects indicated that the normal QTVI and ${\mathrm{QT}}_{\text{SD}}$ values were –1.6 and 3.3 ms, respectively; meanwhile, in ischemic disease, these values grew to –0.6 and 7.3 ms, respectively [20]. Finally, in CHF patients, the QTVI increases because of aging [18, 22, 23], the NYHA class [85], systolic function reduction [18], left ventricular hypertrophy [91] and sympathetic over-activation [87], but it has been reduced during β adrenergic block [25, 92, 93]. In particular, in an experimental study, an increase in the QTVI during sympathetic stimulation only in congestive HF, but not in normal conditions, was observed [25]. Although the QTVI and ${\mathrm{QT}}_{\text{SD}}$ have been studied to identify patients with a high risk of malignant ventricular arrhythmias and sudden cardiac death, these two electrical markers have never entered clinical practice nor even specific scientific society guidelines. However, previously, a large prospective study called “Muerte Subita en Insuficiencia Cardiaca” was able to demonstrate that the QTVI was predictive for cardiovascular mortality but not for sudden cardiac death in patients with CHF [93]. In other words, the QTVI should be considered more as a nonspecific marker of cardiovascular mortality rather than a noninvasive electrophysiologic risk marker of sudden cardiac death. More recently, researchers’ attention has been focused on the last part of ECG myocardial repolarization instead of the entire QT interval. In fact, many authors have studied the total and sudden cardiovascular mortality risk related to the T peak–T end interval duration (Tpe), that is, the interval between the peak and the end of the T wave [94, 95], even in animal models [96]. In particular, in 2016, some authors published a large retrospective study in almost 140,000 individuals over fifty years old in the Copenhagen area, regarding mortality and Tpe duration [97]. In this study, the authors reported for subjects with a Tpe between 110 and 140 ms (95th percentile) a higher risk for all-cause mortality, cardiovascular death, atrial fibrillation, and HF. In addition, in a meta-analysis, conducted on 33 studies and in almost 160,000 subjects, the authors reported that a prolongation of a Tpe higher than 103 ms was a predictive marker not only for malignant ventricular arrhythmias and sudden and non-sudden cardiac death but also for all-cause mortality [98]. Finally, our group recently observed that the longer the Tpe in decompensated CHF subjects, the higher the in-hospital and 30-day mortality was [86, 87, 89]. In particular, the value of Tpe was obtained as the mean of the Tpe intervals in 5-minute EGG recordings. Moreover, in the abovementioned studies, a value of Tpe equal to or greater than 116 ms was a risk factor for 30 days total and cardiovascular mortality [85]. Obviously, in the Copenhagen study [97] and in the T peak–end’s length meta-analysis [98], the standard measurement of Tpe was calculated on a small number of QRS-T complexes. However, with 5-minute ECG recordings, it was possible to calculate the dispersion of the short-term temporal variability of the Tpe intervals, also reported as the standard deviation of the mean (${\mathrm{Tpe}}_{\text{SD}}$). Therefore, the ${\mathrm{Tpe}}_{\text{SD}}$ was significantly related to the NT-proBNP (r: 0.471; *p* < 0.001) in decompensated CHF subjects [88, 90]. In conclusion, Tpe could be also considered a mortality marker that is more accurate than the QT or RR variability, and the ${\mathrm{Tpe}}_{\text{SD}}$ could be a marker of decompensation that could be used as a surrogate for NT-proBNP in the point of care, especially in a remote position, such as at home or on the way to the hospital (Fig. 6).

## 3. Remote Hemodynamic Monitoring

The possibility to remotely monitor the hemodynamic status of CHF patients was studied with different implantable devices, and it was also offered as an optional feature in pace-makers, resynchronization therapy, and implantable converter defibrillator devices. Nevertheless, the usefulness of these devices is still debated regarding the potential mortality risk reduction; on the contrary, they seemed more useful to individuate an increased risk of rehospitalization [99, 100].

Historically, many studies have investigated the possibility of using intrathoracic bioimpedance measurements as a method of evaluating patients at high risk for exacerbation of heart failure or cardiovascular events over time [101, 102, 103]. The fundamental assumption, underlying the remote monitoring of CHF patients, is that before decompensation and rehospitalization, a period from days to weeks occurs in which the left ventricular end-diastolic or filling pressure increases [104]. In other words, by optimizing the therapy, rehospitalizations and re-exacerbations could be avoided [105]. Thus, how to obtain a measure of the left ventricular end-diastolic pressure to anticipate the symptoms of decompensation is an open issue. At present, these parameters are invasively acquired. During the last two decades, three different possible invasive technological solutions have been developed. The first device was able to continuously monitor the right ventricular pressure with an implantable sensor; the pressure in this cardiac chamber is considered a surrogate for PAWP and LVEDP. This device, similar to a pacemaker and named the Chronicle implantable hemodynamic monitor (IHM) (Medtronic, Inc, Minneapolis, Minnesota), can be subcutaneously implanted in the pectoral muscle area. It is provided with a lead with a pressure sensor near the tip and placed in the right ventricular outflow tract or septum. A randomized prospective single-blind parallel trial was conducted to prove the efficacy of this device; this study was named the Chronicle Offers Management to Patients with Advanced Sign and Symptoms of Heart Failure (COMPASS-HF) [106]. The major aim of this study was to prove if the Chronicle IHM in CHF patients provided hemodynamic information useful for optimizing the therapy and reducing mortality and rehospitalization risk. The trial was conducted in 274 centers, enrolling III/IV NYHA class CHF patients, and the follow-up was a period of six months [106]. This trial reported a reduction of 21% of HF-related events (hospitalization, emergency, or urgent visits requiring intravenous therapy), but this result was not statistically significant in comparison to the control group. However, a retrospective analysis demonstrated a significant reduction of 36% of rehospitalization in the group with activated Chronicle IHM. The group with activated Chronicle IHM had 28% more adjusted therapies in comparison to the control group. We also believe that some severe limitations, such as, for example, the incompatibility with atrial pacing, cardiac resynchronization therapy, and magnetic resonance imaging induced early obsolescence of this device.

A second, more advanced device was developed to directly and remotely monitor the pulmonary artery pressures and to guide the therapy; the Cardio-Microelectromechanical system (CardioMEMS) (Abbott, Sylmar, CA, USA) can be implanted via the femoral vein during a right cardiac catheterization [105, 106]. Once implanted, this pulmonary wireless sensor is externally powered by means of radiofrequency energy, and it was designed to be permanently placed in the pulmonary artery. CardioMEMS was studied in a large single-blind randomized trial named the US CardioMEMS Heart Sensor Allows Monitoring of Pressure to Improve Outcomes in NYHA Class III Heart Failure Patients (US CHAMPION). A total of 550 III NYHA class patients were enrolled in this trial, and the primary end-point was the rate of rehospitalization, checked at an 18-month follow-up. Although the US CHAMPION did not demonstrate a reduction in mortality, the rehospitalization rate was significantly reduced by 37% [107, 108]. Furthermore, during the following open-label period, this device showed an additional reduction in the rehospitalization rate (48%) [109]. Finally, the CardioMEMS was approved by both the Food and Drug Administration and the European Conformity. US and European authors are studying a new device similar to the CardioMEMS, but with more hemodynamic parameters, named the ${\mathrm{Cordella}}^{\text{TM}}$ (Endotronix Inc, Chicago, IL, USA) [110], but data from randomized clinical trials are not available yet [111].

Obviously, pulmonary pressure is not only related to pulmonary congestion and the left ventricular end-diastolic pressure but also to the pulmonary pre-capillary resistance; for this reason, a sensor was designed to directly measure the pressure in the left atrium.

In a recent paper [112], the authors emphasized the need to study the longitudinal cardiovascular pressure–volume relationships in the dynamic clinical environment of HF. Their findings do indicate that pressure-based assessment of congestion in ambulatory HF patients does not accurately represent intravascular volume; nevertheless, they assumed that the pressure changes remain indicative of HF exacerbations. Additional volume-based phenotyping may be required to guide decongestion strategies in patients with HF. There is initial evidence that patients with a low or normal volume (independent of peripheral arterial disease (PAD)) are at the greatest risk for HF hospitalization [112]. Therefore, the third technology solution involves the implantation of a sensor in the left atrium directly. The HeartPOD system (Abbot, formerly St. Jude Medical/Savacor, Inc.Abbott Parl, IL, USA) is a sensor designed to be implanted in the left atrium and to measure its pressures [113]. This invasive device is implanted via the femoral vein and by trans-septal puncture. It is powered and measured by a wireless advisory module through the skin. The results on its safety were disappointing because the Left Atrium Pressure Monitoring to Optimize Heart Failure Therapy (LAPTOP-HF) study, designed to evaluate its efficacy in guiding medical therapy, was interrupted early due to an excess of procedure-related complications [114]. Despite this interruption, the results were evaluated in the remaining 486 patients during twelve months of follow-up, and the HeartPOD was capable of significantly reducing the rehospitalization rate by 41% [114].

All in all, the possibility of monitoring the pressure in the left atrium has certainly brought clinical advantages beyond the problems of implantation. In fact, the next generation of studied implantable devices is the V-LAP (Vectorious Medical Technologies, Tel Aviv, Israel), and it could represent a new tool for left atrium pressure monitoring [115]. At present, a trial on its safety, usability, and efficacy is still ongoing. Among the physiologic parameters, the V-LAP will take into account the heart rate variability and a new generation of decision-support software [116]. Obviously, the data unavailability in elderly and fragile patients, which is the category of subjects most affected by CHF, remains a critical issue. Thus, ethical or rational issues could arise proposing a diagnostic invasive device to a fragile patient with many comorbidities.

A second critical point is represented by the device costs. A cost-effectiveness analysis was published on the CardioMEMS using the CHAMPION trial data. The CardioMEMS showed an increase in the cost-effective ratio in comparison to a standard approach also using the five-year outcome data. In particular, the CardioMEMS cost-effective ratio was USD 44,832 per quality-adjusted life-year higher than the standard cure [117].

Currently, noninvasive measurement of the left ventricular end-diastolic pressure is impracticable, but bioimpedance cardiography could allow the noninvasive evaluation of hemodynamic parameters (stroke volume and cardiac output) and tele-monitoring should be possible. The bioimpedance is based on the calculation of the systolic and diastolic difference of electrical impedance by cutaneous leads; in fact, the thoracic water volume changes during the cardiac cycle and it is able to influence the electrical impedance. Obviously, the electrical neck-thorax impedance difference is related to the systolic and diastolic ventricular volumes, so, by appropriate algorithms, the stroke volume (SV) and other hemodynamic noninvasive derived data could be collected [118, 119]. Nowadays, domestic hemodynamic self-monitoring by bioimpedance cardiography devices is not possible and only available for medical clinical research purposes [120]. In this era, the biomedical technology industries could easily develop a simple device for the self-monitoring of hemodynamic parameters so that the patients or their caregivers could easily learn to place six skin leads (two on the left base of the neck—supraclavicular fossa, two at the midpoint of the thorax, and two for the ECG), recording their own hemodynamic data via a smartphone [118, 119]. For example, the PhysioFlow (Manatec Biomedical, Poissy, France) is able to provide an interesting noninvasive systolic and diastolic hemodynamic dataset [121, 122]. The systolic parameters are the SV, SV index (Svi), cardiac output (CO), cardiac index (CI), systemic vascular resistance (SVR), systemic vascular resistance index (SVRi), ejection fraction (EF), contractility index (CTI), left ventricular ejection time (LVET), and left cardiac work index (LCWi). Moreover, the diastolic parameters are the LVEDV and early diastolic filling ratio (EDFR). Briefly, the second derivative of impedance (dZ/dt) measures the temporal variation of impedance, and it is acquired for the calculation of the SV with the following formula: SV = *k*ꞏ[(${\mathrm{dZ/dt}}_{\text{max}}$)/($Z_{\text{max}}$ – $Z_{\text{min}}$)]ꞏW (thoracic flow inversion ${\mathrm{time}}_{\text{cal}}$), where the *k* is a constant, W is a propriety correction algorithm, and cal indicates that the value was obtained during autocalibration [111]. The CO is calculated as SV⋅ heart rate (${\mathrm{Lꞏmin}}^{-1}$), and the SVR as 80⋅(mean blood pressure-central pressure)/CO (${\mathrm{dynꞏs}}^{-1}$⋅${\mathrm{cm}}^{5}$). The central venous pressure is, by default, set as 7 mmHg. Obviously, the CO, SV, and SVR are indexed for the body surface area, and they become CI, Svi, and SVRi, respectively. The VET is the time between the opening and closing aortic valve (ms) from the dZ/dt. The EF is calculated according to the Caplan formula [123]: EF = 0.84 – (0.64ꞏpre-ejection period)/left ventricular ejection time (LVET) (%). The pre-ejection period is the intervals obtained from the Q wave (ECG) and the opening of the aortic valve. The LCWi is calculated as LCWi = 0.0144⋅CI⋅ (mean blood pressure – pulmonary artery occlusion pressure) (kg⋅$m^{-1}$⋅$m^{2}$). The pulmonary artery occlusion is set as 10 mmHg by default. The CTI is calculated with the following formula: CTI = ${\mathrm{dZ/dt}}_{\text{peak}}$.

**Fig. 7.**
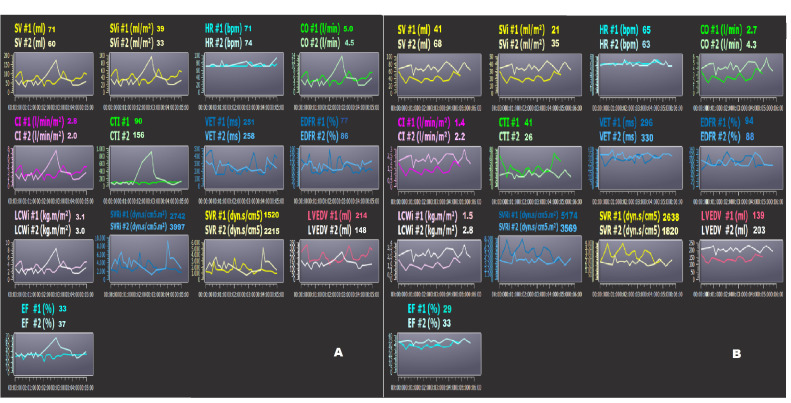
**Bioimpedence PhysioFlow analysis**. (A,B) two patients’ bioimpedance data (PhysioFlow) at the time of admission (#1) and discharge (#2) to the hospital. Note the patient in Panel A had decreased LVEDV, but the same subject showed an increase in SVRi and decrease in CI. The patient in Panel B had increased LVEDV at the time of discharge. In this subject, the risk of decompensation could be higher. SV, stroke volume; SVi, stroke volume index; CO, cardiac output; CI, cardiac index; SVR, systemic vascular resistance; EF, ejection fraction; CTI, contractility index; VET, left ventricular ejection time; LCWi, left cardiac work index; LVEDV, left ventricular end-diastolic volume; EDFR, early diastolic filling ratio; SVRi, systemic vascular resistance index; HR, heart rate.

Finally, the diastolic parameters are the diastolic end diastolic volume (EDV) and the EDFR. The LVEDV is calculated as SV/EF (mL), and the EDFR is obtained by the dZ/dt as the ratio between the diastolic and systolic waves [118, 124]. The EDFR is correlated positively to the integral of the Doppler echocardiographic A wave and negatively to age [125]. Two examples of noninvasive monitoring by bioimpedance (PhysioFlow) in two HFrEF hospitalized patients are reported in Fig. 7A,B, at the time of admission (#1) and discharge (#2) to the hospital. The patient (Fig. 7A) reduced the LVEDV by 31%; this datum is confirmed by the reduction of the NT-proBNP by 55% (from 2910 to 1320 pg/mL). On the other hand, the same patient showed an increase in the SVRi and a decrease in the CI (Fig. 7A). The other patient (Fig. 7B) had an increased LVEDV by 41%, despite an increase in drug therapy, at the time of hospital discharge. The same subject did not have any changes in the NT-proBNP blood level in comparison to the moment of admission. Probably, this patient could have had a higher risk for future decompensation. Obviously, bioimpedance cardiography could never replace the more complex or invasive methods but could be used to monitor patients in remote positions (at home or in a nursing home) after discharge. For example, our preliminary data indicated that the LVEDV obtained in hypertensive subjects with a PhysioFlow and standard echocardiography are significantly related (n.: 19; r: 0.676; *p*: 0.003) [118].

Finally, some other techniques should be taken into account in order to better quantify the decompensation degree of CHF patients. Remote dielectric sensing (ReDS) has been proposed for the telemonitoring of CHF patients [126]. ReDS, which is a novel noninvasive wearable device to quantify the degree of pulmonary congestion easily and quickly within a minute, would be a promising supportive tool to guide titration of the dose of diuretics in patients with congestive heart failure in outpatient clinical follow-up. ReDS detects acute heart failure similar to the lung ultrasound score and primarily identifies the acute heart failure patients who have congestion on a chest Computer Tomography [126, 127].

## 4. Conclusions

The continuous aging of the population and the recent pandemic have highlighted the increasing need to promote health and quality of life, particularly for patients affected by chronic diseases. People and healthcare professionals should increase awareness and knowledge about chronic heart failure burden in the healthcare systems. An approach specifically tailored to the patient’s characteristics (age, sex/gender, comorbidities) must be improved. The possibility of remotely monitoring chronic patients should be applied to all chronic heart failure subjects in order to guide therapeutic choices and avoid exacerbations. Noninvasive technologies specifically suitable for elderly and fragile patients with CHF are not widely available yet. Detection at the point of care of humoral biomarkers and electrical signals able to select subjects with a higher risk of CHF exacerbations should be pursued. Future research should aim towards this new frontier and beyond the challenges of the coming years.

## References

[1] van Riet EES, Hoes AW, Wagenaar KP, Limburg A, Landman MAJ, Rutten FH (2016). Epidemiology of heart failure: the prevalence of heart failure and ventricular dysfunction in older adults over time. A systematic review. *European Journal of Heart Failure*.

[2] Rey JR, Caro-Codón J, Rosillo SO, Iniesta ÁM, Castrejón-Castrejón S, Marco-Clement I (2020). Heart failure in COVID-19 patients: prevalence, incidence and prognostic implications. *European Journal of Heart Failure*.

[3] Arrigo M, Jessup M, Mullens W, Reza N, Shah AM, Sliwa K (2020). Acute heart failure. *Nature Reviews: Disease Primers*.

[4] McDonagh TA, Metra M, Adamo M, Gardner RS, Baumbach A, Böhm M (2021). 2021 ESC Guidelines for the diagnosis and treatment of acute and chronic heart failure. *European Heart Journal*.

[5] Bozkurt B, Coats AJS, Tsutsui H, Abdelhamid CM, Adamopoulos S, Albert N (2021). Universal definition and classification of heart failure: a report of the Heart Failure Society of America, Heart Failure Association of the European Society of Cardiology, Japanese Heart Failure Society and Writing Committee of the Universal Definition of Heart Failure: Endorsed by the Canadian Heart Failure Society, Heart Failure Association of India, Cardiac Society of Australia and New Zealand, and Chinese Heart Failure Association. *European Journal of Heart Failure*.

[6] Hunt SA, Abraham WT, Chin MH, Feldman AM, Francis GS, Ganiats TG (2005). ACC/AHA 2005 Guideline Update for the Diagnosis and Management of Chronic Heart Failure in the Adult: a report of the American College of Cardiology/American Heart Association Task Force on Practice Guidelines (Writing Committee to Update the 2001 Guidelines for the Evaluation and Management of Heart Failure): developed in collaboration with the American College of Chest Physicians and the International Society for Heart and Lung Transplantation: endorsed by the Heart Rhythm Society. *Circulation*.

[7] (1994). *The Criteria Committee of the New York Heart Association. Nomenclature and Criteria for Diagnosis of Diseases of the Heart and Great Vessels. 9th edn*.

[8] Mascherbauer J, Zotter-Tufaro C, Duca F, Binder C, Koschutnik M, Kammerlander AA (2017). Wedge Pressure Rather Than Left Ventricular End-Diastolic Pressure Predicts Outcome in Heart Failure With Preserved Ejection Fraction. *JACC: Heart Failure*.

[9] Piccirillo G, Magrì D, di Carlo S, De Laurentis T, Torrini A, Matera S (2006). Influence of cardiac-resynchronization therapy on heart rate and blood pressure variability: 1-year follow-up. *European Journal of Heart Failure*.

[10] Zucker IH, Patel KP, Schultz HD (2012). Neurohumoral stimulation. *Heart Failure Clinics*.

[11] Piccirillo G, Luparini RL, Celli V, Moisè A, Lionetti M, Marigliano V (2000). Effects of carvedilol on heart rate and blood pressure variability in subjects with chronic heart failure. *The American Journal of Cardiology*.

[12] (1996). Heart rate variability: standards of measurement, physiological interpretation and clinical use. Task Force of the European Society of Cardiology and the North American Society of Pacing and Electrophysiology. *Circulation*.

[13] Bigger JT, Fleiss JL, Steinman RC, Rolnitzky LM, Kleiger RE, Rottman JN (1992). Frequency domain measures of heart period variability and mortality after myocardial infarction. *Circulation*.

[14] La Rovere MT, Bigger JT, Marcus FI, Mortara A, Schwartz PJ (1998). Baroreflex sensitivity and heart-rate variability in prediction of total cardiac mortality after myocardial infarction. ATRAMI (Autonomic Tone and Reflexes After Myocardial Infarction) Investigators. *The Lancet*.

[15] Huikuri HV, Mäkikallio TH, Peng CK, Goldberger AL, Hintze U, Møller M (2000). Fractal correlation properties of R-R interval dynamics and mortality in patients with depressed left ventricular function after an acute myocardial infarction. *Circulation*.

[16] Hayano J, Kiyono K, Struzik ZR, Yamamoto Y, Watanabe E, Stein PK (2011). Increased non-gaussianity of heart rate variability predicts cardiac mortality after an acute myocardial infarction. *Frontiers in Physiology*.

[17] La Rovere MT, Pinna GD, Maestri R, Mortara A, Capomolla S, Febo O (2003). Short-term heart rate variability strongly predicts sudden cardiac death in chronic heart failure patients. *Circulation*.

[18] Piccirillo G, Rossi P, Mitra M, Quaglione R, Dell’Armi A, Di Barba D (2013). Indexes of temporal myocardial repolarization dispersion and sudden cardiac death in heart failure: any difference. *Annals of Noninvasive Electrocardiology*.

[19] Piccirillo G, Magnanti M, Matera S, Di Carlo S, De Laurentis T, Torrini A (2006). Age and QT variability index during free breathing, controlled breathing and tilt in patients with chronic heart failure and healthy control subjects. *Translational Research*.

[20] Baumert M, Porta A, Vos MA, Malik M, Couderc JP, Laguna P (2016). QT interval variability in body surface ECG: measurement, physiological basis, and clinical value: position statement and consensus guidance endorsed by the European Heart Rhythm Association jointly with the ESC Working Group on Cardiac Cellular Electrophysiology. *Europace*.

[21] Piccirillo G, Magrì D, Matera S, Magnanti M, Torrini A, Pasquazzi E (2007). QT variability strongly predicts sudden cardiac death in asymptomatic subjects with mild or moderate left ventricular systolic dysfunction: a prospective study. *European Heart Journal*.

[22] Piccirillo G, Magrì D, Matera S, Magnanti M, Pasquazzi E, Schifano E (2008). Effects of pink grapefruit juice on QT variability in patients with dilated or hypertensive cardiomyopathy and in healthy subjects. *Translational Research*.

[23] Piccirillo G, Moscucci F, Pascucci M, Pappadà MA, D’Alessandro G, Rossi P (2013). Influence of aging and chronic heart failure on temporal dispersion of myocardial repolarization. *Clinical Interventions in Aging*.

[24] Piccirillo G, Cacciafesta M, Lionetti M, Nocco M, Di Giuseppe V, Moisè A (2001). Influence of age, the autonomic nervous system and anxiety on QT-interval variability. *Clinical Science*.

[25] Piccirillo G, Ogawa M, Song J, Chong VJ, Joung B, Han S (2009). Power spectral analysis of heart rate variability and autonomic nervous system activity measured directly in healthy dogs and dogs with tachycardia-induced heart failure. *Heart Rhythm*.

[26] Ogawa M, Zhou S, Tan AY, Song J, Gholmieh G, Fishbein MC (2007). Left stellate ganglion and vagal nerve activity and cardiac arrhythmias in ambulatory dogs with pacing-induced congestive heart failure. *Journal of the American College of Cardiology*.

[27] Shen MJ, Zipes DP (2014). Role of the autonomic nervous system in modulating cardiac arrhythmias. *Circulation Research*.

[28] Fonarow GC, Abraham WT, Albert NM, Stough WG, Gheorghiade M, Greenberg BH (2008). Factors identified as precipitating hospital admissions for heart failure and clinical outcomes: findings from OPTIMIZE-HF. *Archives of Internal Medicine*.

[29] Arrigo M, Gayat E, Parenica J, Ishihara S, Zhang J, Choi DJ (2017). Precipitating factors and 90-day outcome of acute heart failure: a report from the intercontinental GREAT registry. *European Journal of Heart Failure*.

[30] Kommuri NVA, Koelling TM, Hummel SL (2012). The impact of prior heart failure hospitalizations on long-term mortality differs by baseline risk of death. *The American Journal of Medicine*.

[31] Solomon SD, Dobson J, Pocock S, Skali H, McMurray JJV, Granger CB (2007). Influence of nonfatal hospitalization for heart failure on subsequent mortality in patients with chronic heart failure. *Circulation*.

[32] Raffaello WM, Henrina J, Huang I, Lim MA, Suciadi LP, Siswanto BB (2021). Clinical Characteristics of De Novo Heart Failure and Acute Decompensated Chronic Heart Failure: Are They Distinctive Phenotypes That Contribute to Different Outcomes. *Cardiac Failure Review*.

[33] Feldman DS, Elton TS, Sun B, Martin MM, Ziolo MT (2008). Mechanisms of disease: detrimental adrenergic signaling in acute decompensated heart failure. *Nature Clinical Practice. Cardiovascular Medicine*.

[34] Redfield MM, Chen HH, Borlaug BA, Semigran MJ, Lee KL, Lewis G (2013). Effect of phosphodiesterase-5 inhibition on exercise capacity and clinical status in heart failure with preserved ejection fraction: a randomized clinical trial. *The Journal of the American Medical Association*.

[35] O’Connor CM, Starling RC, Hernandez AF, Armstrong PW, Dickstein K, Hasselblad V (2011). Effect of nesiritide in patients with acute decompensated heart failure. *The New England Journal of Medicine*.

[36] Pfisterer M, Buser P, Rickli H, Gutmann M, Erne P, Rickenbacher P (2009). BNP-guided vs symptom-guided heart failure therapy: the Trial of Intensified vs Standard Medical Therapy in Elderly Patients With Congestive Heart Failure (TIME-CHF) randomized trial. *The Journal of the American Medical Association*.

[37] Piccirillo G, Moscucci F, Carnovale M, Corrao A, Di Diego I, Lospinuso I (2022). Short-Period Temporal Dispersion Repolarization Markers in Elderly Patients with Decompensated Heart Failure. *La Clinica Terapeutica*.

[38] Murphy SP, Kakkar R, McCarthy CP, Januzzi JL (2020). Inflammation in Heart Failure: JACC State-of-the-Art Review. *Journal of the American College of Cardiology*.

[39] Gallego-Colon E, Bonaventura A, Vecchié A, Cannatà A, Fitzpatrick CM (2020). Cardiology on the cutting edge: updates from the European Society of Cardiology (ESC) Congress 2020. *BMC Cardiovascular Disorders*.

[40] Crousillat DR, Ibrahim NE (2018). Sex Differences in the Management of Advanced Heart Failure. *Current Treatment Options in Cardiovascular Medicine*.

[41] Han Z, Chen Z, Lan R, Di W, Li X, Yu H (2017). Sex-specific mortality differences in heart failure patients with ischemia receiving cardiac resynchronization therapy. *PLoS ONE*.

[42] Shuaishuai D, Jingyi L, Zhiqiang Z, Guanwei F (2022). Sex differences and related estrogenic effects in heart failure with preserved ejection fraction. *Heart Failure Reviews*.

[43] Cesaroni G, Mureddu GF, Agabiti N, Mayer F, Stafoggia M, Forastiere F (2021). Sex differences in factors associated with heart failure and diastolic left ventricular dysfunction: a cross-sectional population-based study. *BMC Public Health*.

[44] Geraghty L, Figtree GA, Schutte AE, Patel S, Woodward M, Arnott C (2021). Cardiovascular Disease in Women: From Pathophysiology to Novel and Emerging Risk Factors. *Heart, Lung & Circulation*.

[45] Scicchitano P, Paolillo C, De Palo M, Potenza A, Abruzzese S, Basile M (2022). Sex Differences in the Evaluation of Congestion Markers in Patients with Acute Heart Failure. *Journal of Cardiovascular Development and Disease*.

[46] Yerly A, van der Vorst EPC, Baumgartner I, Bernhard SM, Schindewolf M, Döring Y (2023). Sex-specific and hormone-related differences in vascular remodelling in atherosclerosis. *European Journal of Clinical Investigation*.

[47] de Miguel-Balsa E (2022). Risk stratification and health inequalities in women with acute coronary syndrome: time to move on. *The Lancet*.

[48] Wenzl FA, Kraler S, Ambler G, Weston C, Herzog SA, Räber L (2022). Sex-specific evaluation and redevelopment of the GRACE score in non-ST-segment elevation acute coronary syndromes in populations from the UK and Switzerland: a multinational analysis with external cohort validation. *The Lancet*.

[49] Sciomer S, Moscucci F, Salvioni E, Marchese G, Bussotti M, Corrà U (2020). Role of gender, age and BMI in prognosis of heart failure. *European Journal of Preventive Cardiology*.

[50] Ahmad J, Ahmad HA, Surapaneni P, Penagaluri A, Desai S, Dominic P (2022). Women are underrepresented in cardiac resynchronization therapy trials. *Journal of Cardiovascular Electrophysiology*.

[51] Goda A, Lund LH, Mancini D (2011). The Heart Failure Survival Score outperforms the peak oxygen consumption for heart transplantation selection in the era of device therapy. *The Journal of Heart and Lung Transplantation*.

[52] Levy WC, Mozaffarian D, Linker DT, Sutradhar SC, Anker SD, Cropp AB (2006). The Seattle Heart Failure Model: prediction of survival in heart failure. *Circulation*.

[53] Sartipy U, Dahlström U, Edner M, Lund LH (2014). Predicting survival in heart failure: validation of the MAGGIC heart failure risk score in 51,043 patients from the Swedish heart failure registry. *European Journal of Heart Failure*.

[54] Agostoni P, Corrà U, Cattadori G, Veglia F, La Gioia R, Scardovi AB (2013). Metabolic exercise test data combined with cardiac and kidney indexes, the MECKI score: a multiparametric approach to heart failure prognosis. *International Journal of Cardiology*.

[55] Salvioni E, Corrà U, Piepoli M, Rovai S, Correale M, Paolillo S (2020). Gender and age normalization and ventilation efficiency during exercise in heart failure with reduced ejection fraction. *ESC Heart Failure*.

[56] Corrà U, Agostoni P, Giordano A, Cattadori G, Battaia E, La Gioia R (2016). Sex Profile and Risk Assessment With Cardiopulmonary Exercise Testing in Heart Failure: Propensity Score Matching for Sex Selection Bias. *The Canadian Journal of Cardiology*.

[57] Son YJ, Won MH (2020). Gender differences in the impact of health literacy on hospital readmission among older heart failure patients: A prospective cohort study. *Journal of Advanced Nursing*.

[58] Defilippis EM, Truby LK, Clerkin KJ, Donald E, Sinnenberg L, Varshney AS (2022). Increased Opportunities for Transplantation for Women in the New Heart Allocation System. *Journal of Cardiac Failure*.

[59] Moscucci F, Lavalle F, Politi C, Campanale A, Baggio G, Sciomer S (2022). Acute coronary syndrome in women: a new and specific approach is needed. *European Journal of Preventive Cardiology*.

[60] Nadar SK, Shaikh MM (2019). Biomarkers in Routine Heart Failure Clinical Care. *Cardiac Failure Review*.

[61] Rubattu S, Triposkiadis F (2017). Resetting the neurohormonal balance in heart failure (HF): the relevance of the natriuretic peptide (NP) system to the clinical management of patients with HF. *Heart Failure Reviews*.

[62] Shrivastava A, Haase T, Zeller T, Schulte C (2020). Biomarkers for Heart Failure Prognosis: Proteins, Genetic Scores and Non-coding RNAs. *Frontiers in Cardiovascular Medicine*.

[63] Weber M, Hamm C (2006). Role of B-type natriuretic peptide (BNP) and NT-proBNP in clinical routine. *Heart*.

[64] Iwanaga Y, Nishi I, Furuichi S, Noguchi T, Sase K, Kihara Y (2006). B-type natriuretic peptide strongly reflects diastolic wall stress in patients with chronic heart failure: comparison between systolic and diastolic heart failure. *Journal of the American College of Cardiology*.

[65] Maisel AS, Krishnaswamy P, Nowak RM, McCord J, Hollander JE, Duc P (2002). Rapid measurement of B-type natriuretic peptide in the emergency diagnosis of heart failure. *The New England Journal of Medicine*.

[66] Januzzi JL, Camargo CA, Anwaruddin S, Baggish AL, Chen AA, Krauser DG (2005). The N-terminal Pro-BNP investigation of dyspnea in the emergency department (PRIDE) study. *The American Journal of Cardiology*.

[67] Heidenreich PA, Bozkurt B, Aguilar D, Allen LA, Byun JJ, Colvin MM (2022). 2022 AHA/ACC/HFSA Guideline for the Management of Heart Failure: Executive Summary: A Report of the American College of Cardiology/American Heart Association Joint Committee on Clinical Practice Guidelines. *Journal of the American College of Cardiology*.

[68] Anwaruddin S, Lloyd-Jones DM, Baggish A, Chen A, Krauser D, Tung R (2006). Renal function, congestive heart failure, and amino-terminal pro-brain natriuretic peptide measurement: results from the ProBNP Investigation of Dyspnea in the Emergency Department (PRIDE) Study. *Journal of the American College of Cardiology*.

[69] Peacock WF, De Marco T, Fonarow GC, Diercks D, Wynne J, Apple FS (2008). Cardiac troponin and outcome in acute heart failure. *The New England Journal of Medicine*.

[70] Xue Y, Clopton P, Peacock WF, Maisel AS (2011). Serial changes in high-sensitive troponin I predict outcome in patients with decompensated heart failure. *European Journal of Heart Failure*.

[71] Ather S, Hira RS, Shenoy M, Fatemi O, Deswal A, Aguilar D (2013). Recurrent low-level troponin I elevation is a worse prognostic indicator than occasional injury pattern in patients hospitalized with heart failure. *International Journal of Cardiology*.

[72] deFilippi CR, de Lemos JA, Christenson RH, Gottdiener JS, Kop WJ, Zhan M (2010). Association of serial measures of cardiac troponin T using a sensitive assay with incident heart failure and cardiovascular mortality in older adults. *The Journal of the American Medical Association*.

[73] De Vecchis R, Ariano C (2016). Measuring B-Type Natriuretic Peptide From Capillary Blood or Venous Sample: Is It the Same. *Cardiology Research*.

[74] Sörensen NA, Neumann JT, Ojeda F, Giannitsis E, Spanuth E, Blankenberg S (2019). Diagnostic Evaluation of a High-Sensitivity Troponin I Point-of-Care Assay. *Clinical Chemistry*.

[75] Hight M, Conklin K, Archer B, Sutherland J, Sakai B, Arnold D (2021). Implementing Point-of-Care Troponin Testing in the Emergency Department: Impact on Time to Result. *Journal of Emergency Nursing*.

[76] Apple FS, Collinson PO, Kavsak PA, Body R, Ordóñez-Llanos J, Saenger AK (2021). Getting Cardiac Troponin Right: Appraisal of the 2020 European Society of Cardiology Guidelines for the Management of Acute Coronary Syndromes in Patients Presenting without Persistent ST-Segment Elevation by the International Federation of Clinical Chemistry and Laboratory Medicine Committee on Clinical Applications of Cardiac Bio-Markers. *Clinical Chemistry*.

[77] Serhani MA, T El Kassabi H, Ismail H, Nujum Navaz A (2020). ECG Monitoring Systems: Review, Architecture, Processes, and Key Challenges. *Sensors*.

[78] (1996). Heart rate variability. Standards of measurement, physiological interpretation, and clinical use. Task Force of the European Society of Cardiology and the North American Society of Pacing and Electrophysiology. *European Heart Journal*.

[79] Aronson D, Burger AJ (2001). Effect of beta-blockade on heart rate variability in decompensated heart failure. *International Journal of Cardiology*.

[80] Aronson D, Burger AJ (2001). Effect of beta-blockade on autonomic modulation of heart rate and neurohormonal profile in decompensated heart failure. *Annals of Noninvasive Electrocardiology*.

[81] Piccirillo G, Magrì D, Naso C, di Carlo S, MoisE A, De Laurentis T (2004). Factors influencing heart rate variability power spectral analysis during controlled breathing in patients with chronic heart failure or hypertension and in healthy normotensive subjects. *Clinical Science*.

[82] Piccirillo G, Moscucci F, D’Alessandro G, Pascucci M, Rossi P, Han S (2014). Myocardial repolarization dispersion and autonomic nerve activity in a canine experimental acute myocardial infarction model. *Heart Rhythm*.

[83] Magrì D, De Cecco CN, Piccirillo G, Mastromarino V, Serdoz A, Muscogiuri G (2014). Myocardial repolarization dispersion and late gadolinium enhancement in patients with hypertrophic cardiomyopathy. *Circulation Journal*.

[84] Haigney MC, Zareba W, Gentlesk PJ, Goldstein RE, Illovsky M, McNitt S (2004). QT interval variability and spontaneous ventricular tachycardia or fibrillation in the Multicenter Automatic Defibrillator Implantation Trial (MADIT) II patients. *Journal of the American College of Cardiology*.

[85] Berger RD, Kasper EK, Baughman KL, Marban E, Calkins H, Tomaselli GF (1997). Beat-to-beat QT interval variability: novel evidence for repolarization lability in ischemic and nonischemic dilated cardiomyopathy. *Circulation*.

[86] Piccirillo G, Moscucci F, Bertani G, Lospinuso I, Sabatino T, Zaccagnini G (2021). Short-period temporal repolarization dispersion in subjects with atrial fibrillation and decompensated heart failure. *Pacing and Clinical Electrophysiology*.

[87] Piccirillo G, Moscucci F, Iorio CD, Fabietti M, Mastropietri F, Crapanzano D (2020). Time- and frequency-domain analysis of repolarization phase during recovery from exercise in healthy subjects. *Pacing and Clinical Electrophysiology*.

[88] Piccirillo G, Moscucci F, Bertani G, Lospinuso I, Mastropietri F, Fabietti M (2020). Short-Period Temporal Dispersion Repolarization Markers Predict 30-Days Mortality in Decompensated Heart Failure. *Journal of Clinical Medicine*.

[89] Piccirillo G, Moscucci F, Mariani MV, Di Iorio C, Fabietti M, Mastropietri F (2020). Hospital mortality in decompensated heart failure. A pilot study. *Journal of Electrocardiology*.

[90] Magrì D, Piccirillo G, Bucci E, Pignatelli G, Cauti FM, Morino S (2012). Increased temporal dispersion of myocardial repolarization in myotonic dystrophy type 1: beyond the cardiac conduction system. *International Journal of Cardiology*.

[91] Piccirillo G, Germanò G, Quaglione R, Nocco M, Lintas F, Lionetti M (2002). QT-interval variability and autonomic control in hypertensive subjects with left ventricular hypertrophy. *Clinical Science*.

[92] Piccirillo G, Quaglione R, Nocco M, Naso C, Moisè A, Lionetti M (2002). Effects of long-term beta-blocker (metoprolol or carvedilol) therapy on QT variability in subjects with chronic heart failure secondary to ischemic cardiomyopathy. *The American Journal of Cardiology*.

[93] Tereshchenko LG, Cygankiewicz I, McNitt S, Vazquez R, Bayes-Genis A, Han L (2012). Predictive value of beat-to-beat QT variability index across the continuum of left ventricular dysfunction: competing risks of noncardiac or cardiovascular death and sudden or nonsudden cardiac death. *Circulation. Arrhythmia and Electrophysiology*.

[94] Tse G, Yan BP (2017). Traditional and novel electrocardiographic conduction and repolarization markers of sudden cardiac death. *Europace*.

[95] Piccirillo G, Moscucci F, Fabietti M, Di Iorio C, Mastropietri F, Sabatino T (2020). Age, gender and drug therapy influences on Tpeak-tend interval and on electrical risk score. *Journal of Electrocardiology*.

[96] Piccirillo G, Magrì D, Pappadà MA, Maruotti A, Ogawa M, Han S (2012). Autonomic nerve activity and the short-term variability of the Tpeak-Tend interval in dogs with pacing-induced heart failure. *Heart Rhythm*.

[97] Bachmann TN, Skov MW, Rasmussen PV, Graff C, Pietersen A, Lind B (2016). Electrocardiographic Tpeak-Tend interval and risk of cardiovascular morbidity and mortality: Results from the Copenhagen ECG study. *Heart Rhythm*.

[98] Tse G, Gong M, Wong WT, Georgopoulos S, Letsas KP, Vassiliou VS (2017). The Tpeak - Tend interval as an electrocardiographic risk marker of arrhythmic and mortality outcomes: A systematic review and meta-analysis. *Heart Rhythm*.

[99] Imberti JF, Tosetti A, Mei DA, Maisano A, Boriani G (2021). Remote monitoring and telemedicine in heart failure: implementation and benefits. *Current Cardiology Reports*.

[100] Wang L, Lahtinen S, Lentz L, Rakow N, Kaszas C, Ruetz L (2005). Feasibility of using an implantable system to measure thoracic congestion in an ambulatory chronic heart failure canine model. *Pacing and Clinical Electrophysiology*.

[101] Packer M, Abraham WT, Mehra MR, Yancy CW, Lawless CE, Mitchell JE (2006). Utility of impedance cardiography for the identification of short-term risk of clinical decompensation in stable patients with chronic heart failure. *Journal of the American College of Cardiology*.

[102] Castellanos LR, Bhalla V, Isakson S, Daniels LB, Bhalla MA, Lin JP (2009). B-type natriuretic peptide and impedance cardiography at the time of routine echocardiography predict subsequent heart failure events. *Journal of Cardiac Failure*.

[103] Adamson PB (2009). Pathophysiology of the transition from chronic compensated and acute decompensated heart failure: new insights from continuous monitoring devices. *Current Heart Failure Reports*.

[104] Radhoe SP, Veenis JF, Brugts JJ (2021). Invasive Devices and Sensors for Remote Care of Heart Failure Patients. *Sensors*.

[105] Bourge RC, Abraham WT, Adamson PB, Aaron MF, Aranda JM, Magalski A (2008). Randomized controlled trial of an implantable continuous hemodynamic monitor in patients with advanced heart failure: the COMPASS-HF study. *Journal of the American College of Cardiology*.

[106] Abraham WT, Adamson PB, Bourge RC, Aaron MF, Costanzo MR, Stevenson LW (2011). Wireless pulmonary artery haemodynamic monitoring in chronic heart failure: a randomised controlled trial. *The Lancet*.

[107] Abraham WT, Stevenson LW, Bourge RC, Lindenfeld JA, Bauman JG, Adamson PB (2016). Sustained efficacy of pulmonary artery pressure to guide adjustment of chronic heart failure therapy: complete follow-up results from the CHAMPION randomised trial. *The Lancet*.

[108] Desai AS, Bhimaraj A, Bharmi R, Jermyn R, Bhatt K, Shavelle D (2017). Ambulatory Hemodynamic Monitoring Reduces Heart Failure Hospitalizations in “Real-World” Clinical Practice. *Journal of the American College of Cardiology*.

[109] Mullens W, Sharif F, Dupont M, Rothman AMK, Wijns W (2020). Digital health care solution for proactive heart failure management with the Cordella Heart Failure System: results of the SIRONA first-in-human study. *European Journal of Heart Failure*.

[110] Guichard JL, Cowger JA, Chaparro SV, Kiernan MS, Mullens W, Mahr C (2023). Rationale and Design of the Proactive-HF Trial for Managing Patients With NYHA Class III Heart Failure by Using the Combined Cordella Pulmonary Artery Sensor and the Cordella Heart Failure System. *Journal of Cardiac Failure*.

[111] Yaranov DM, Jefferies JL, Silver MA, Burkhoff D, Rao VN, Fudim M (2022). Discordance of Pressure and Volume: Potential Implications for Pressure-Guided Remote Monitoring in Heart Failure. *Journal of Cardiac Failure*.

[112] Ritzema J, Melton IC, Richards AM, Crozier IG, Frampton C, Doughty RN (2007). Direct left atrial pressure monitoring in ambulatory heart failure patients: initial experience with a new permanent implantable device. *Circulation*.

[113] Maurer MS, Adamson PB, Costanzo MR, Eigler N, Gilbert J, Gold MR (2015). Rationale and Design of the Left Atrial Pressure Monitoring to Optimize Heart Failure Therapy Study (LAPTOP-HF). *Journal of Cardiac Failure*.

[114] Miyagi C, Kuroda T, Karimov JH, Fukamachi K (2022). Novel approaches for left atrial pressure relief: Device-based monitoring and management in heart failure. *Frontiers in Cardiovascular Medicine*.

[115] Abraham WT, Perl L (2017). Implantable Hemodynamic Monitoring for Heart Failure Patients. *Journal of the American College of Cardiology*.

[116] Schmier JK, Ong KL, Fonarow GC (2017). Cost-Effectiveness of Remote Cardiac Monitoring With the CardioMEMS Heart Failure System. *Clinical Cardiology*.

[117] Anand G, Yu Y, Lowe A, Kalra A (2021). Bioimpedance analysis as a tool for hemodynamic monitoring: overview, methods and challenges. *Physiological Measurement*.

[118] Piccirillo G, Moscucci F, Corrao A, Carnovale M, Di Diego I, Lospinuso I (2022). Noninvasive Hemodynamic Monitoring in Advanced Heart Failure Patients: New Approach for Target Treatments. *Biomedicines*.

[119] Kemps HMC, Thijssen EJM, Schep G, Sleutjes BTHM, De Vries WR, Hoogeveen AR (2008). Evaluation of two methods for continuous cardiac output assessment during exercise in chronic heart failure patients. *Journal of Applied Physiology*.

[120] Gordon N, R Abbiss C, J Maiorana A, J Marston K, J Peiffer J (2018). Intrarater Reliability And Agreement Of The Physioflow Bioimpedance Cardiography Device During Rest, Moderate And High-Intensity Exercise. *Kinesiology*.

[121] Lewicki L, Fijalkowska M, Karwowski M, Siebert K, Redlarski G, Palkowski A (2021). The non-invasive evaluation of heart function in patients with an acute myocardial infarction: The role of impedance cardiography. *Cardiology Journal*.

[122] van der Meer NJ, Oomen MW, Vonk Noordegraaf A, Pijpers RJ, Plaizier MA, de Vries PM (1996). Does impedance cardiography reliably estimate left ventricular ejection fraction. *Journal of Clinical Monitoring*.

[123] Leão RN, Silva PMD (2019). Impedance Cardiography in the Evaluation of Patients with Arterial Hypertension. *International Journal of Cardiovascular Sciences*.

[124] Pickett BR, Buell JC (1993). Usefulness of the impedance cardiogram to reflect left ventricular diastolic function. *The American Journal of Cardiology*.

[125] Imamura T, Kinugawa K (2022). Clinical insight of remote dielectric sensing-guided congestive heart failure management in outpatient clinic. *Journal of Cardiology Cases*.

[126] Olesen ASO, Miger K, Fabricius-Bjerre A, Sandvang KD, Kjesbu IE, Sajadieh A (2022). Remote dielectric sensing to detect acute heart failure in patients with dyspnoea: a prospective observational study in the emergency department. *European Heart Journal Open*.

[127] Faragli A, Abawi D, Quinn C, Cvetkovic M, Schlabs T, Tahirovic E (2021). The role of non-invasive devices for the telemonitoring of heart failure patients. *Heart Failure Reviews*.

